# Evidence of introduced honeybees (*Apis mellifera*) as pollen wasters in orchid pollination

**DOI:** 10.1038/s41598-024-64218-x

**Published:** 2024-06-18

**Authors:** Daniela Scaccabarozzi, Lorenzo Guzzetti, Emiliano Pioltelli, Mark Brundrett, Andrea Aromatisi, Giovanni Polverino, Mario Vallejo-Marin, Salvatore Cozzolino, Zong-Xin Ren

**Affiliations:** 1 Department of Ecology and Genetics, Evolutionary Biology Centre, Uppsala University, Norbyvägen 18 D, 752 36 Uppsala, Sweden; 2 School of Molecular and Life Sciences, Curtin University, Perth, Australia; 3 ZooPlantLab, Department of Biotechnology and Biosciences, University of Milano-Bicocca, Milan, Italy; 4 School of Biological Sciences, University of Western Australia, Crawley, Australia; 5 Earth to Be, Consulting Group, Perth, Australia; 6 Department of Ecological and Biological Sciences, University of Tuscia, Viterbo, Italy; 7 Department of Biology, University of Naples Federico II, Naples, Italy; 8 Kunming Institute of Botany, Chinese Academy of Sciences (CAS), Kunming, China

**Keywords:** Habitat alteration, Introduced honeybees, Invasive species, Orchids, Pollination, Native bees, Ecology, Evolution

## Abstract

Biological invasions threaten global biodiversity, altering landscapes, ecosystems, and mutualistic relationships like pollination. Orchids are one of the most threatened plant families, yet the impact of invasive bees on their reproduction remains poorly understood. We conduct a global literature survey on the incidence of invasive honeybees (*Apis mellifera*) on orchid pollination, followed by a study case on Australian orchids. Our literature survey shows that *Apis mellifera* is the primary alien bee visiting orchids worldwide. However, in most cases, introduced honeybees do not deposit orchid pollen. We also test the extent to which introduced honeybees affect orchid pollination using *Diuris brumalis* and *D. magnifica*. *Diuris brumalis* shows higher fruit set and pollination in habitats with both native and invasive bees compared to habitats with only introduced bees. Male and female reproductive success in *D. magnifica* increases with native bee abundance, while conversely pollinator efficiency decreases with honeybee abundance and rises with habitat size. Our results suggest that introduced honeybees are likely involved in pollen removal but do not effectively deposit orchid pollen, acting as pollen wasters. However, *Apis mellifera* may still contribute to pollination of *Diuris* where native bees no longer exist. Given the global occurrence of introduced honeybees, we warn that certain orchids may suffer from pollen depletion by these invaders, especially in altered habitats with compromised pollination communities.

## Introduction

Biological invasions are one of the leading threats to global biodiversity^1^, impacting the structure and dynamics of landscapes, communities, and ecosystems^2^. The cascading effects of alien species can adversely affect mutualistic relationships among plant and animals, including pollination^2^. Particularly, invasive bees can change the original plant-pollinator networks and even harm both plant and pollinator partners^3^. By competing with native pollinators for floral resources and nesting sites^4–6^, invasive bees can impact pollinator fitness and population dynamics^7–9^. Through altering pollen flow, alien pollinators are in general expected to compromise plant reproductive success^10^, limit pollen availability to native pollinators^2,10,11^ and increase heterospecific pollen deposition^2,12^.

European honeybees (*Apis mellifera*) have become principal floral visitors of plant species of ecosystems around the world^13^, but their effect on plant reproductive success is complex to detect^14^ and to assess^3^. Honeybees are generalist pollinators and frequent plant visitors but may not necessarily benefit plant reproduction of all species^15^, especially when they competitively replace native pollinators and become ineffective surrogates^16^. Conversely, in cases where native pollinators are rare or locally extinct, honeybees often boost pollination^17,18^ or can even recover plant fitness from reproductive collapse in fragmented habitat^19^. However, most studies have documented how honeybees impact native bee communities through floral resource competition, whilst their effect on plant reproduction remains poorly documented^3,14^.

Orchids present highly specialised pollination mechanisms and given the great adaptability of honeybees to floral resources, the impact of invasive honeybees on the fitness of these plants might be important. Studies evaluating the effect of introduced *Apis mellifera* on orchid pollination success are very scarce. Beyond their renowned diversity of pollination systems, orchids can attract pollinators with nonrewarding flowers via various modes of deception^20–24^. About 46% of all orchid species globally are thought to lack reward^25,26^, typically resulting in lower insect visitation rates compared to rewarding ones^27,28^, deserving careful consideration for their conservation biology. Given that orchids offer pollen in discrete pollinia, instead of unpacked pollen grains as occurs in most other flowering plants, it is even more important to maximise the pollen transfer and deposition among flowers during pollinator visits^29^. A measure of the effectiveness of pollen transfer is pollination efficiency (PE) that is typically measured as the ratio of pollinated flowers on flowers with pollinia removed^30,31^. During transfer by pollinators, pollen losses in orchids are expected to be high when mediated by generalist pollinators and by a range of pollinator types^27,32^. For these reasons, pollinator efficiency in orchids might be hampered by exotic and generalist honeybees that manage to collect the pollinia but are not morphologically configurated to successfully deposit the pollinia and guarantee reproduction of the plant. Whilst in most cases pollinia removal and fruit set are similar across populations^33–35^, in some orchid species these trends can diverge. For example, the food deceptive Australian orchid species *Diuris brumalis* shows diverse responses of male and female reproductive success in relation to model plants’ abundance, with the first according to an exponential growth and the second to a logarithmic growth^36^. This variation can be attributed to the improved ‘learning behaviour’ of bees that have encountered deceptive orchids before and removed pollinia, so these are more likely to distinguish deceptive orchids from model plants^4,37^. As a result, the success of deception may stabilise with more abundance of rewarding plants^36^.

Our overall objective is to understand how introduced honeybees impact orchid pollination. We address this objective using (a) a literature search, to identify how often honeybees remove and deposit pollinia from orchid flowers, and (b) a case study which compares pollination success and efficiency of orchids in sites where native bee pollinators and honeybees co-occur and sites where native bee pollinators were fully replaced by invasive honeybees.

**Figure 1:**
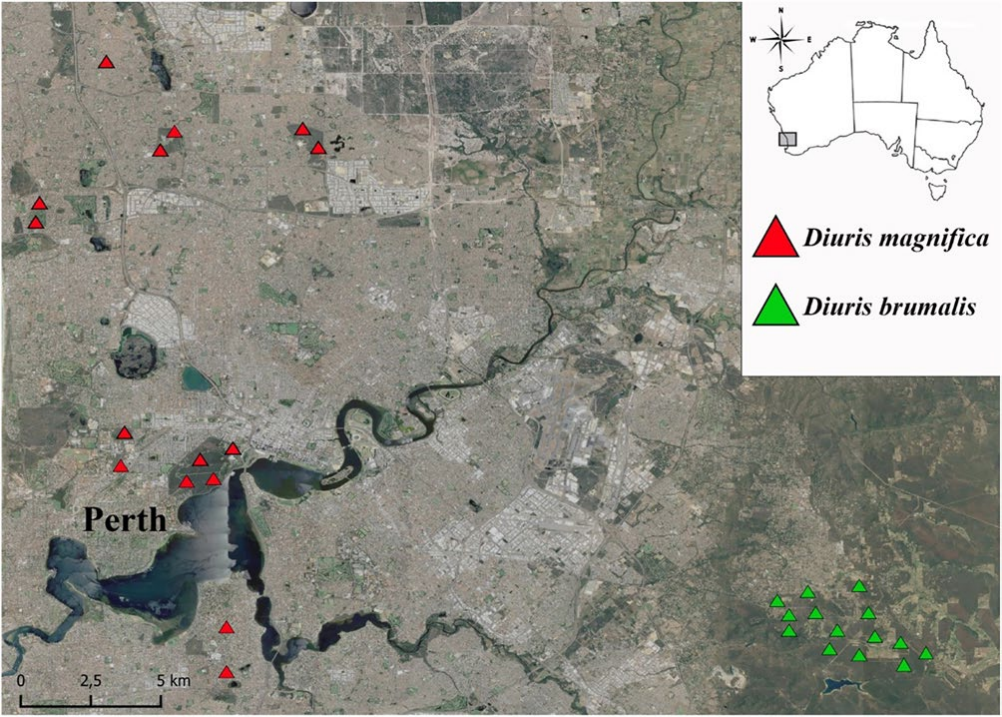
Sites where populations of *Diuris brumalis* (red triangles) and *D. magnifica* (green triangles) were studied in the metropolitan area of Perth and Perth hills respectively, Southwestern Australia. Geographic coordinates are reported in Datafile S1. Satellite imagery was obtained from Google Maps, and the map was created using QGIS 3.10 (2019) - QGIS Geographic Information System. Open-Source Geospatial Foundation Project: http://qgis.org.

**Figure 2:**
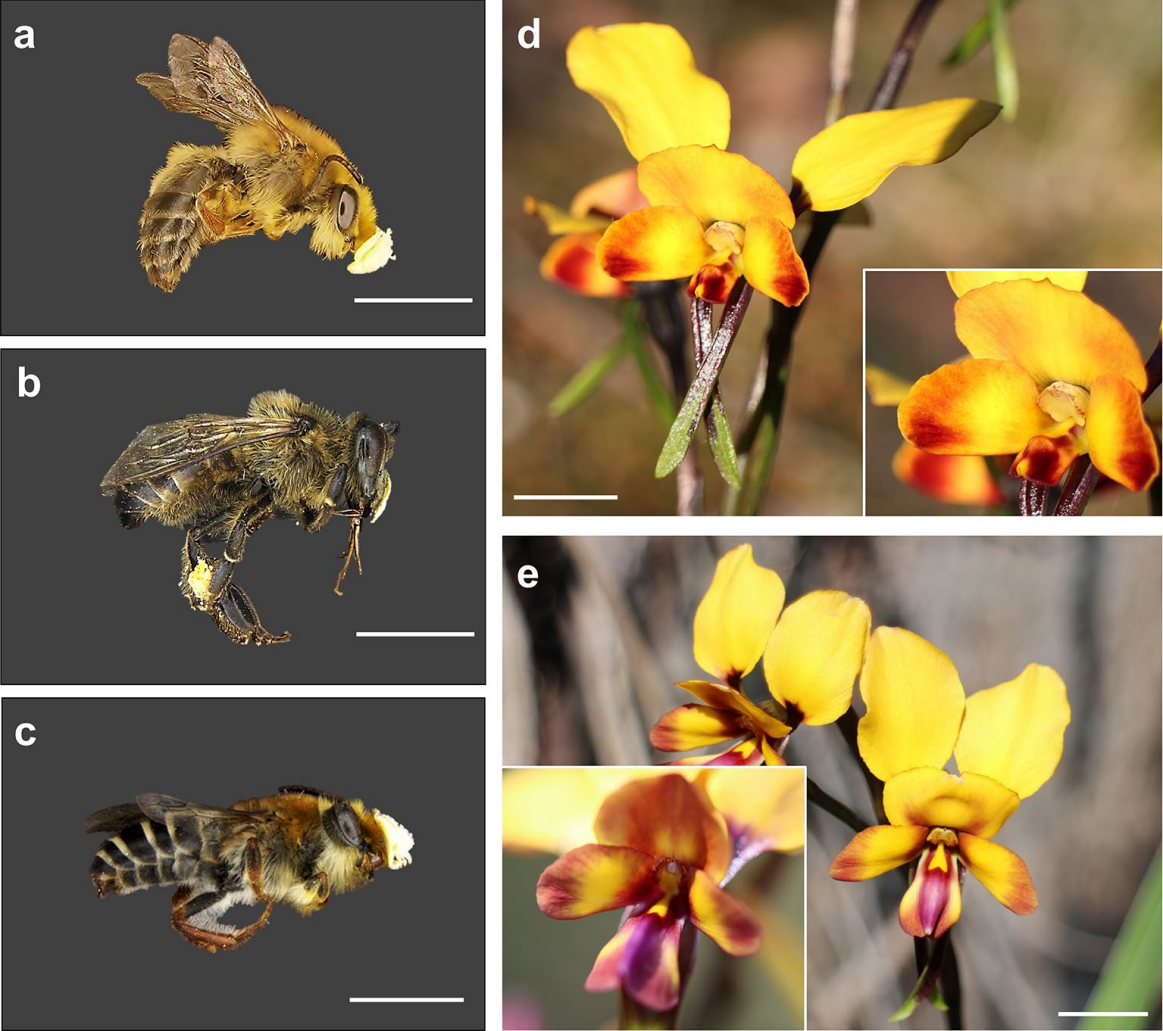
Native and non-native pollinators carrying *Diuris* (Orchidaceae) pollinia. Pollinia placement on *Trichocolletes capillosus*, native pollinator for *Diuris brumalis* (**a**), on *Apis mellifera*, pollen remover for *D. brumalis* and *D. magnifica* (**b**); and on *Trichocolletes gelasinus*, native pollinator of *D. magnifica* (**c**); flower morphology of *D. brumalis* (**d**) and* D. magnifica* (**e**) flowers showing the column where the pollinia is placed at the top and the labellum, insect platform. Scale bar of 5 mm. Credit: Daniela Scaccabarozzi.

To conduct our empirical study, we focus on two orchid species in the genus *Diuris* (Orchidaceae) with analogous pollination strategies (food deception) and occupying different habitats that are subjected to different levels of human alteration (Fig. 1). As *D. brumalis* and *D. magnifica* populations inhabit sites subjected to anthropogenic alteration, we hypothesise that in disturbed sites lacking native pollinators^36^, alien honeybees can act as surrogates for pollinia removal but not for pollen transfer. Both species are generally pollinated by native bees of the genus *Trichocolletes* and are occasionally visited by the introduced *Apis mellifera* that potentially acts as a sub-optimal pollinator^29,36^ (Fig. 2). Whilst *Apis mellifera* is ubiquitous in all study sites, native bees (*Trichocolletes*) are patchily distributed across the sites. Given that pollination success for *D. brumalis* varies according to habitat type (forest *vs* disturbed woodland)^36^ and is also related to habitat size (*Banksia* woodland) for *D. magnifica*^38^, we hypothesise that the presence and abundance of native and exotic pollinators alter pollination success and efficiency in response to these habitat conditions.

For *D. brumalis*, we expect that: (i) the presence of native pollinators and exotic bees varies according to habitat type (forest *vs* disturbed woodland); (ii) removal of pollinia does not differ in sites where native pollinators occur relative to sites where native pollinators are absent; (iii) fruit set and pollen efficiency are higher in sites with native pollinators. For *D. magnifica*, we expect that: (i) removal of pollinia, fruit set and PE increase with the abundance of native pollinators; (ii) PE decreases with the abundance of introduced honeybees; (iii) and PE also decreases with habitat size.

## Results

### Literature review: incidence of honeybees in pollination of orchids

A total of 124 publications were included in the literature survey, covering 120 different orchid species overall (see Table 1, Table S1, Fig. S1) that were visited by honeybees, either observed to remove pollinaria (potential pollination) or pollinate flowers (depositing pollinia). These included all continents where orchids occur. Europe represented the 36% of total cases, followed by Asia (34%), America (14%), Oceania (12%) and Africa (0.04%) (Fig. S2). Of the total number of orchid species visited, pollinated, or potentially pollinated by honeybees, introduced honeybees represented 32% of cases. Pollination or pollinaria removal by introduced bees was recorded most often for the Epidendroideae (46%), followed by Orchidoideae subfamily (31%), Vanilloideae (17%) and Cypripedioideae (0.06%), as expected given the size of each subfamily. The introduced honeybee (*Apis mellifera*) was observed to act as a: visitor (V, when only observed landing on a flower) in 15% of cases; a pollen depositor (PD, when successfully depositing a pollinia at least once) in 31% of cases or pollen remover (PR, when removing pollinia at least once) in 54% of cases. Of the total number of orchid species visited by honeybees, honeybees were non-native in 32% of cases. In a few cases, *A. mellifera* was accompanied by other introduced bee genera such as *Bombus*, *Centris* and *Euglossa*.

**Table 1:** Literature survey presenting the incidence of non-native species *Apis mellifera* (Apidae) in orchid pollination across continents (species by alphabetic order), according to the following described categories: V: visitor; PR: pollen remover; PD: pollen depositor; n.a.: not available.

Continent	Country	Subfamily	Plant species	Native or alien plant species	Native bee or other native pollinators	Introduced bee species	Pollination by introduced species	Literature source
America	Puerto Rico	Epidendroideae	*Arundina graminifolia*	Native	*Megachile yaeyamaensi, Thyreus takaonis*	*Apis mellifera* (Africanized honeybee)	PR	Ackerman^39^; Sugiura^40^
Asia	Japan, South Korea	Epidendroideae	*Bletilla striata*	Native	likely *Tetralonia nipponensis*	*Apis mellifera* (Africanized honeybee)	PD	Sugiura^41^; Ogawa and Miyake^42^
America	Chile, Argentina Andes	Orchidoideae	*Brachystele unilateralis*	Native	*Bombus dahlbomii*	*Apis mellifera, Bombus terrestris, Bombus ruderarius*	PD	Sanguinetti and Singer^43^
Oceania	Western Australia	Orchidoideae	*Caladenia flava*	Native	*Neophyllotocus,* native bee	*Apis mellifera*	V	Adams and Lawson^44^; Fig S1 and Daniela Scaccabarozzi personal observation
Oceania	Western Australia	Orchidoideae	*Caladenia xantha*	Native	n.a	*Apis mellifera*	V	Photo Mark Brundrett, Brundrett et al.^45^; Fig. S1
America	North America	Epidendroideae	*Calopogon pallidus*	Native	*Bombus* sp., *Xylocopa virginica*	*Apis mellifera*	PD	Luer^46^; Argue^47^
America	North America	Epidendroideae	*Calopogon tuberosus*	Native	*Bombus americanorum, B. grisecollis, B. vagans, B. fervidus B. nevadensis, B. ternarius, B. terricola, Xylocopa virginica, X. micans, Augochlora * sp., *Megachile melanophea*	*Apis mellifera*	PD	Luer^46^; Thien and Marcks^48^; Heinrich^49^
America	Chile, Argentina Andes	Orchidoideae	*Chloraea virescens*	Native	*Bombus dahlbomii*	*Apis mellifera, Bombus terrestris, Bombus ruderarius*	PD	Sanguinetti and Singer^43^
America	North America	Vanilloideae	*Cleistesiopsis divaricata*	Native	*Megachile * sp, *Bombus pennsylvanicus, B. fervidus, B. bimaculatus, B. vagans, B. impatiens, B. bimaculatus*	*Apis mellifera*	PD	Gregg^50,51^
Asia	India	Epidendroideae	*Cymbidium pendulum*	Native	*Apis cerana*	*Apis mellifera*	PD	Attri and Kant^52^; Verma et al.^53^
Asia	Japan	Cypripedioideae	*Cypripedium macranthos*	Native	*Andrena ruficrus, Bombus pseudobaicaiensis*	*Apis mellifera*	PR	Sugiura et al.^54,55^
America	USA	Cypripedioideae	*Cypripedium candidum*	Native	likely *Andrena sp*., *Odontomyia pubescens* (Diptera)	*Apis mellifera*	PR	Pearn^56^; Grantham et al.^57^
America	USA	Cypripedioideae	*Cypripedium parviflorum*	Native	likely *Andrena* sp., *Odontomyia pubescens* (Diptera), *Lasioglossum zonulum*	*Apis mellifera*	PR	Pearn^56^; Grantham et al.^57^
America	USA, Canada	Cypripedioideae	*Cypripedium reginae*	Native	likely *Anthophora; Megachile* spp.	*Apis mellifera*	PR	Edens-Meier et al.^58^
America	Mexico	Epidendroideae	*Cyrtopodium macrobulbon*	Native	likely *Centris or Xylocopa*	*Apis mellifera*	PR	Miranda-Molina et al.^59^
Asia	China	Epidendroideae	*Cyrtopodium polyphyllum*	Alien	*Centris tarsata; Centris labrosa*	*Apis mellifera, Centris nitida, Centris errans*	PR	Liu and Pemberton^60^; Pansarin et al.^61^
America	Florida, USA	Epidendroideae	*Cyrtopodium punctatum*	Native	*Xylocopa* sp.	*Apis mellifera, Euglossa dilemma, Centris errans*	V	Pemberton and Liu^62^; Dutra et al.^63^
America	Puerto Rico	Epidendroideae	*Dendrobium crumenatum*	Alien	*Apis cerana*	*Apis mellifera* (Africanized honeybee)	PD	Leong and Wee^64^; Meurgey^65^; Ackerman^66^
Oceania	Eastern Australia	Epidendroideae	*Dendrobium kingianum*	Native	n.a.	*Apis mellifera*	PR	Photo Rudie Kruiter; Fig. S1
Oceania	Australia	Epidendroideae	*Dendrobium speciosum* var.* hillii*	Native	likely *Trigona* sp., *Homalictus* sp*., Lassioglossum, Hylaeus*	*Apis mellifera*	V	Slater and Calder^67^
Oceania	Western Australia	Orchidoideae	*Diuris brumalis*	Native	*Tichocolletes capillosus, Trichocolletes leucogenys*	*Apis mellifera*	PR	Scaccabarozzi et al.^36^
Oceania	Eastern Australia	Orchidoideae	*Diuris maculata*	Native	*Trichocolletes venustus*	*Apis mellifera*	PD	Beardsell et al.^68^; Indsto et al.^69^
Oceania	Western Australia	Orchidoideae	*Diuris magnifica*	Native	*Tichocolletes gelasinus, T.dives*	*Apis mellifera*	PD	Scaccabarozzi et al.^38^
Oceania	Australia	Orchidoideae	*Diuris sulphurea*	Native	*Paracolletes* sp., *Amegilla* sp*.*, *Lipotriches* sp.	*Apis mellifera*	PD	Rayment^70^; Kruiter,^71^
Oceania	Western Australia	Orchidoideae	*Eriochilus dilatatus*	Native	Halictidae bees	*Apis mellifera*	PR	Bundrett^28^; Daniela Scaccabarozzi personal observation
America	Brazil	Orchidoideae	*Ionopsis utricularioides*	Native	Ceratinini, Meliponini, Tapinotaspidini, Halictidae bees	*Apis mellifera scutellata*	V	Aguiar and Pansarin^72^
America	Cayman Islands	Epidendreae	*Myrmecophila thomsoniana*	Native	*Coereba flaveola* (Aves)*, Gymnettis lanius, Lachnopus vanessablockae* (Coleoptera)*, Anolis conspersus* (Reptilia)	*Apis mellifera*	PD	Rose-Smyth^73^
America	North America	Orchidoideae	*Platanthera blephariglottis*	Native	*Bombus fervidus, B. vagans* and various Lepidoptera	*Apis mellifera*	PR	Smith and Snow^74^; Cole and Firmage^75^
Oceania	Australia	Orchidoideae	*Prasophyllum alpinum*	Native	*Pterocormus promissorius* (Ichneumonidae)	*Apis mellifera*	PR	Jones^76^
Oceania	Australia	Orchidoideae	*Prasophyllum elatum*	Native	native bee	*Apis mellifera*	V	Photo Rudie Kruiter; Fig. S1
Oceania	Australia	Orchidoideae	*Prasophyllum* sp.	Native	native bees and wasps	*Apis mellifera*	PR	Photo and personal observation by Rudie Kuiter Fig. S1
Africa	South Africa	Orchidoideae	*Satyrium cristatum*	Native	*Amegilla natalensis, A. spilostoma, A. * sp. *A. natalensis*	*Apis mellifera*	PR	Johnson et al.^77^
Africa	South Africa	Orchidoideae	*Satyrium erectum*	Native	*Anthophora diversipies, A. praecox*	*Apis mellifera*	PR	Ellis and Johnson^78^
Africa	South Africa	Orchidoideae	*Satyrium jacottetiae*	Native	*Philoliche rostrata* (Diptera), *Theretra capensis* (Lepidoptera)	*Apis mellifera*	PD	Botes et al.^79^
Africa	South Africa	Orchidoideae	Schizochilus flexuosus	Native	*Lasioglossum* sp., *Patellapis zonalictus* (Halictidae), Scoliidae	*Apis mellifera*	PR	van der Niet et al.^80^
Oceania	Eastern Australia	Orchidoideae	*Spiranthes australis*	Native	*Amegilla asserta* (likely primary pollinator)	*Apis mellifera*	PR	Ren personal observation; Kuiter^71^
Asia	Japan	Orchidoideae	*Spiranthes australis*	Native	*Megachile nipponica, M. japonica, Halictidae* sp.	*Apis mellifera*	PD	Suetsugu and Abe^81^; Iwata et al.^82^
Oceania	Australia	Orchidoideae	*Spiranthes sinensis*	Native	guild of native bees	*Apis mellifera*	PR	Coleman^83^
America	USA	Orchidoideae	*Spiranthes vernalis*	Native	native bee	*Apis mellifera*	PR	Catling^84^

Personal observations and photos are included to support evidence especially focusing on Australian orchid species. Refer to Table S1 for the literature survey summarising the global incidence of *Apis* bees as a native species.

### Case study on* Diuris brumalis* and* D. magnifica*

#### Pollination in relation to the occurrence of native and alien honeybees

**Figure 3:**
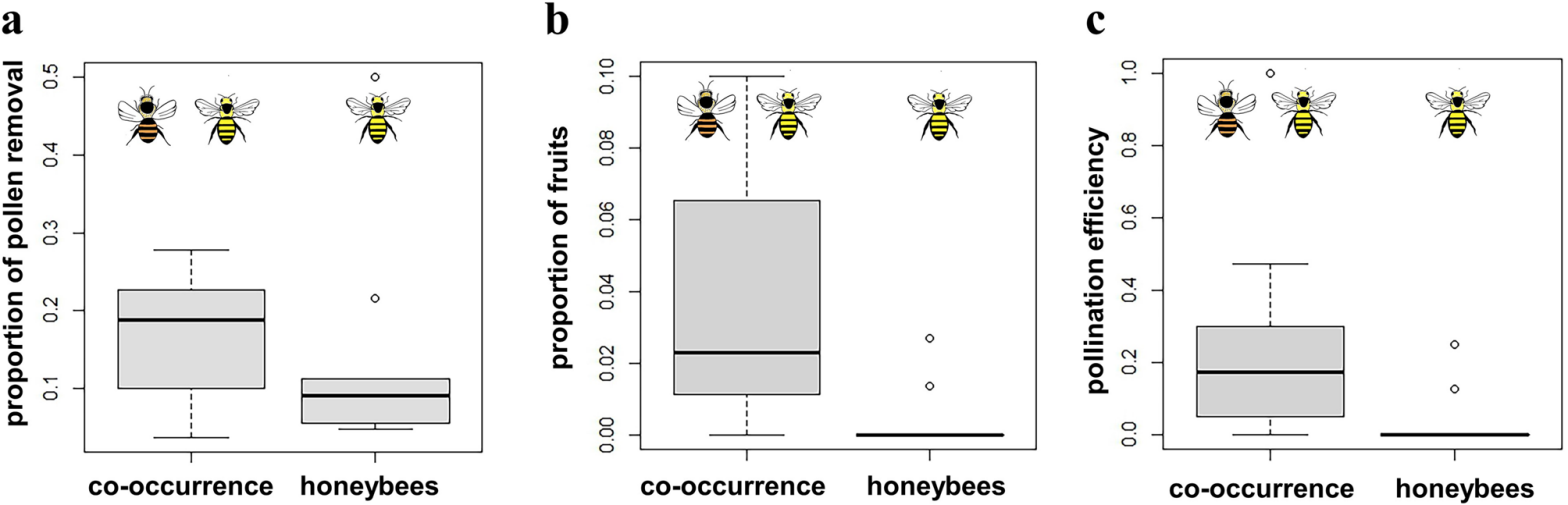
Boxplot of co-occurrence (minimum and maximum value) of honeybees and native bees *vs.* honeybees alone on pollinia removal (**a**), fruit set (**b**), and pollination efficiency (**c**) in *Diuris brumalis* (Orchidaceae).

In D. *brumalis* we found an effect of sampling year on pollinia removal and fruit set. Specifically, the pollinia removal was higher in 2017 (χ2 = 7.4677, *p* = 0.006), whilst fruit set was higher in 2016 (χ2 = 4.6356, *p* = 0.03). Overall pollination efficiency was lower in 2017 than in 2016 (χ2 = 4.1719, p = 0.04). For *D. brumalis*, pollinia removal did not vary between sites characterized by honeybees only and sites with honeybees and native bees co-occurring (Fig. 3a) (χ2 = 2.8637, *p* = 0.091), but sites with only honeybees had significantly lower fruit set (χ2 = 5.4698, *p* = 0.019; Fig. 3b). Pollination efficiency (measured as the proportion of flowers with pollinia removed that also received pollen) was significantly lower where native bees were absent (disturbed woodland) relative to sites with only honeybees (forest) (χ2 = 6.1869, *p* = 0.012) (Fig. 3c).

**Figure 4:**
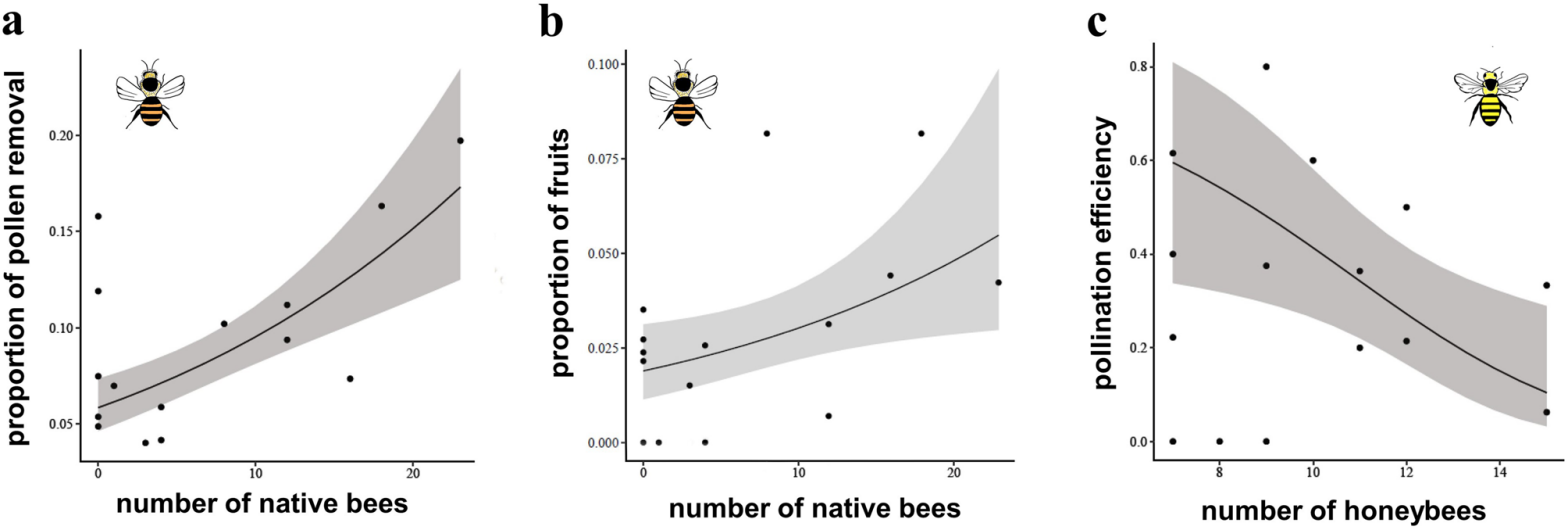
Effect of number of native bees and non-native honeybees quantified during transects for *Diuris magnifica* (Orchidaceae) reproductive success. The number of native bees influences pollinia removal (**a**), and fruit set (**b**) and number of non-native honeybees impacts orchid pollination efficiency (**c**).

In *D. magnifica*, the abundance of native bees was significantly linked to an increase of both pollinia removal (χ2 = 19.572, *p* < 0.001) and fruit set (χ2 = 5.1371, *p* = 0.023) (Fig. 4a,b; Table S2). In particular, the abundance of honeybees led to a decrease of pollination efficiency (χ2 = 7.2195, *p* = 0.007) (Fig. 4 c; Table S2), whilst abundance of native bees did not affect pollination efficiency (Table S2).

#### Effect of habitat type and size on pollination success and efficiency

**Figure 5:**
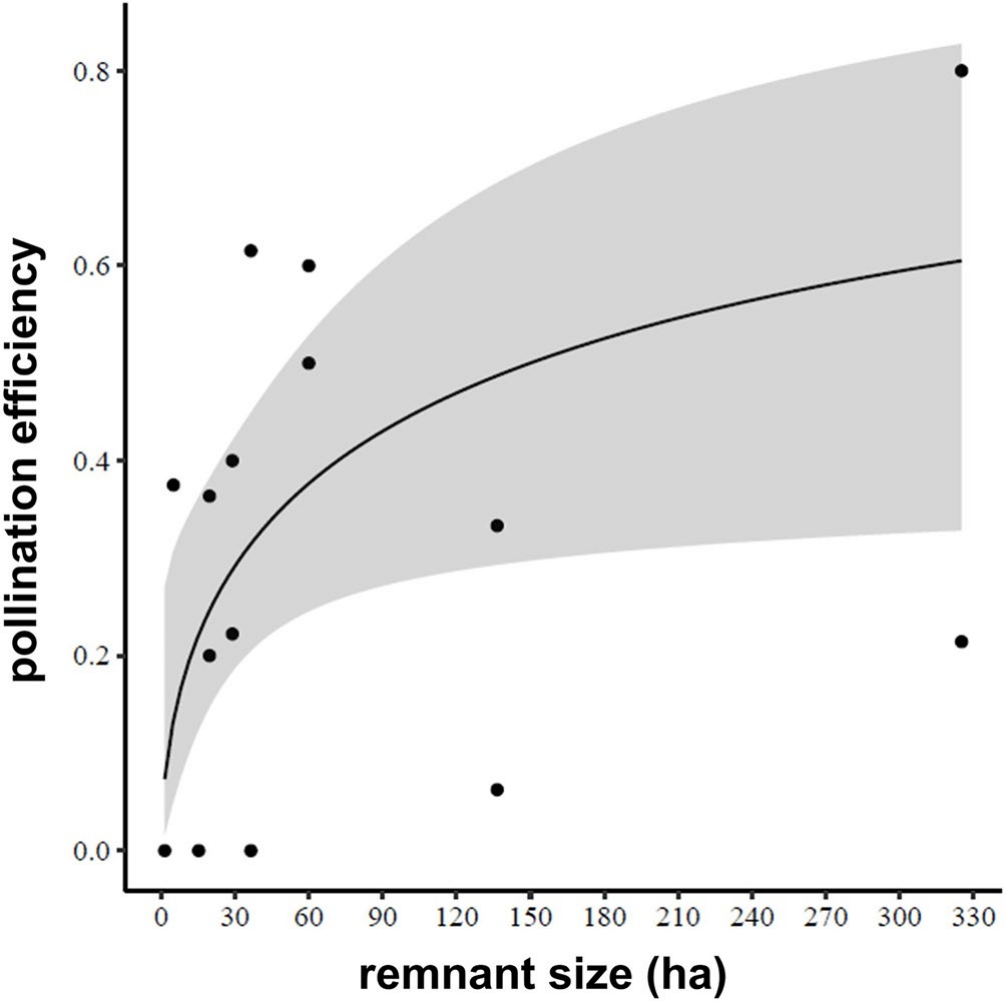
Pollination efficiency of *Diuris magnifica* in relation to bushland reserve area (habitat size).

In *D. brumalis* populations, as native bee pollinator and introduced honeybee occurrence varied by habitat (forest *vs* disturbed habitat) it was not possible to untangle the direct effect of habitat from the other correlated variables. In fact, only honeybees were found in disturbed woodland, whereas honeybees and native bees occurred together in the forest habitat (Fig. 3a–c). In *D. magnifica* populations, habitat remnant size was positively associated with pollination efficiency (χ2 = 6.7399, *p* = 0.009) in a logarithmic manner (Fig. 5).

## Discussion

Our study combined an analysis of experimental data on *Diuris* reproductive success with a literature survey that addresses the role of *Apis* bee species in orchid pollination, focusing on introduced honeybees (*Apis mellifera*). We also examined whether alien honeybees adversely affect pollination success or have the capacity to contribute to orchid pollination in altered landscapes.

The importance of introduced *Apis mellifera*, as a pollinator for orchid species remains unresolved because most studies on interactions between introduced and native bees have focused on other plant families. In our literature survey, *Apis mellifera* is the principal alien bee observed visiting orchids (Table 1). Pollination (or potential pollination) by *Apis* bees (native and introduced) is not common among orchids, resulting in 10% (120 cases in our literature survey) of the ~ 1200 cases of orchid pollination by Hymenoptera^26^. This is a relatively small number compared to the prevalence (~ 60%) of other corbiculate Apidae (including orchid bees and bumblebees) which are specialist pollinators of numerous orchid species^20,26,85,86^ (Table 1 and S1). Despite the widespread distribution of honeybees in Eurasia and Africa^87^, most orchids rely on specific foragers rather than super-generalist pollinators such as honeybees^88^. Even though honeybees are the most frequently observed native pollinator of Mediterranean orchids, no Mediterranean species specialises on *Apis mellifera*^89^. Pollination by introduced *Apis mellifera* accounts for over 3% of the documented cases of orchid pollination by Hymenoptera^86^ and commonly occurs in *Cypripedium*, and *Cyrtopodium* in both Asia and America, in the Australian genus *Diuris*, in *Satyrium* from South Africa and in *Spiranthes* across Australia, Asia and North America (Table1)*.* In these cases, introduced *Apis* are similar in size to at least some of the natural pollinators (i.e., *Amegilla*, *Bombus*, *Megachile*)^90^ (Table 1). In the South American orchid species *Brachystele unilateralis* and *Chloraea virescens*, introduced bees like *Bombus terrestris*, *B. ruderarius* and *Apis mellifera* were successful in displacing the natural pollinator *Bombus dahlbomii*^43^. Therefore, a requisite to be an alien surrogate pollinator seems to be the level of morphological fit between the alien bee and the newly acquired floral resource. According to our review, in two orchid species, the American *Cyrtopodium polyphyllum* and the Asian *Dendrobium crumenatum*, invasive to China and Puerto Rico respectively, introduced species such as *Apis mellifera* acted as pollen removers and pollen depositors respectively^43^. This raises concerns regarding the impact of introduced bees in facilitating the invasion of non-native orchids. Our literature search shows that in most cases introduced *Apis mellifera* have been ineffective in replacing the role of native pollinators. In 69% of recorded cases introduced honeybees were observed as a visitor or a pollen remover but in only 30% of cases they were recorded as a pollinator (pollen depositor).

An important caveat is that, thus far, we have only documented the impact of introduced honeybees on pollinia removal and deposition. However, the generalist foraging behaviour of *Apis mellifera*^15^ may have further implications, including the breakdown of pre-pollination reproductive barriers among coexisting orchid species^15,32,91^. Such hybridization can have evolutionary biological implications for the plant life cycle, the persistence of future generations, diversification, and speciation in Orchidaceae.

In our empirical study, western honeybees occurred in all study sites for both target species (*D. brumalis* and *D. magnifica*) whilst the occurrence of native bees (*Trichocolletes* spp.) was patchy across sites. In *D. brumalis*, honeybees predominantly occurred along with native *Trichocolletes*^36^, but in the absence of native bees, orchid fruit set showed the lowest values at 0% (Fig. 3b). Notably, there was no difference on orchid pollinia removal between sites where honeybees occurred alone (10%) and sites where they co-occurred with native bees (20%) (Fig. 3a), indicating that honeybees led to comparable level of pollinia removal to native bees. Thus, European honeybees are capable of successfully removing pollinia from flowers of *D. brumalis* (Fig. 2a–d), but because fruit set and pollination efficiency were lowest at 0% when honeybees occurred alone, we hypothesise that they deplete pollen supplies available to native pollinators^39^ and are ineffective at pollen deposition. This highlights the value of native pollinator specificity in orchid pollen deposition. According to the *lock* and *key* hypothesis a set of European food deceptive species show higher levels of correlation between pollinarium and stigmatic cavity lengths comparing to sexual deceptive species^92^, to avoid heterospecific pollen deposition of sympatric species. This pollinator specificity seems very crucial in food deceptive species globally^26^.

In *D. magnifica* both pollinia removals and fruit set exponentially increased with native bee abundance (*Trichocolletes gelasinus;* Fig. 4a,b) from 0 to 20% and from 0 to 8% respectively and they were not impacted by the abundance of *Apis mellifera* among study sites. The output was similar among pollinia removal and fruit set and conforms with our expectations that optimal pollinator frequency would enhance orchid reproductive success. Interestingly, in *D. magnifica*, the increase of honeybee abundance reduced the orchid pollination efficiency from 80 to 0% likely because they withdraw pollinia without successfully depositing them on the next flower^3,39^ (Figs. 2b,c,e; 4c) as argued in *D. brumalis*. However, the abundance of native bees did not influence the PE for this species. This could be explained by the patchy occurrence of *Trichocolletes* species across the bushland remnants, especially in smaller bushland reserves. It is also plausible that other factors might interfere with the ability of native pollinators to fulfil their pollination service, i.e., pollinia depletion by honeybees during their visits to the orchids, presence of suboptimal pollinators such as beetles, that were observed to remove pollinia and deposit it on the same flowers on few occasions^38^, and competition between honeybees and wild bees for access to floral resources^3,14^. In addition, plant reproductive success often relies more on bee assemblage and diversity than abundance per se^93^. However, the significant impact of honeybees’ abundance on *D. magnifica* pollination efficiency suggested a detrimental effect of honeybees’ abundance on orchid pollination effectiveness. The honeybee is well known for its modest efficiency in pollination service^21,94^ and in some cases its role may be an antagonistic one where costs (i.e., associated with nectar replenishment, pollen discounting or damage to flowers) exceed the benefits for the plant^95^. One explanation for the potential failure of introduced honeybees in depositing pollen is linked to the mimicry system and the foraging behaviour of native pollinators. *Trichocolletes* bees, the native pollinators of *D. brumalis* and *D. magnifica*, are specialised pollinators of Faboideae species^96^. They are tricked by reward-less orchids via specific floral visual signals that mimic those of Faboideae flowers^97^. In contrast, honeybees, being generalists, visit various flowering plants, including these deceptive orchids, potentially extracting orchid pollinia. However, because they do not exclusively target pea plants like *Trichocolletes* bees, and because orchids do not offer nectar, honeybees are less likely to consistently visit them to deposit pollen. On the other hand, we occasionally observed fruit set of *D. magnifica* and *D. brumalis* in populations where only *Apis mellifera* was present suggesting a local benefit where pollinator networks are compromised (Datafile S1). However, to determine this, assessing seed viability might be necessary. Notably, *Apis mellifera* was observed both removing and depositing pollinia on the same flowers in *D. magnifica*, indicating potential self-pollination rather than pollen transfer between different plants^38^. We suggest that management strategies for beekeeping activities should consider the abundance of alien bees relative to native ones and be designed to reduce antagonistic costs for the plants. We also note that our study sites did not include orchid populations with native bees only, as honeybees have become ubiquitous.

To conclusively test the effect of native bees and introduced honeybees on orchid pollination, and to determine if this effect is influenced by resource overlap between native and introduced bees, it would be necessary to: (i) isolate the effects of native bee occurrence from honeybee occurrence (if feasible); (ii) assess whether the absence of native bees is primarily due to habitat change or competition with honeybees, and (iii) investigate honeybee abundance in intact and altered habitats, respectively.

In our study, habitat type (wild *vs* disturbed) influenced orchid reproductive success in *D. brumalis*, but it was not possible to untangle the direct effect of habitat from the co-occurrence of honeybees and native bees (Fig. 3b,c), because only honeybees occurred in the disturbed woodland site.

We were not able to determine the causes of lack of native pollinators in some study sites, but we hypothesise that anthropogenic habitat alteration (disturbance linked to urban development) might have led to their decline^96,98^. Given that *Trichocolletes* native bees are ground-nesting bees^97^, habitat change might interfere with nesting and foraging sites^4,99,100^, eventually leading to their local loss. This primarily impacts species that employ Batesian floral mimicry such as *D. brumalis* that rely on specialised pollinators^101^.

Our results highlight the importance of conservation of specialised native bee fauna and associated habitats. For *D. magnifica*, larger bushland reserves led to an increase of pollination efficiency (Fig. 4). Specifically, the increase was greatest in the lower half of the predicted trend, where values ranged from 0 to 50% PE and were linked to habitats within a range of 1–60 ha. This means that even relatively small bush fragments can sustain effective pollination service. However, only bigger bushland reserves (over 100 ha) showed PE > 50%, suggesting that the continuous habitat provided more optimal pollination service. This trend might be explained by the expectation that larger habitat sizes sustain a higher biodiversity of native bees (number and richness)^102^.

## Conclusions

Our literature survey highlights the importance of conducting studies on the interaction of native and alien pollinator species globally. Because many members of the orchid family are at high risk of extinction, resolving their pollination ecology in areas occupied by introduced honeybees is vital for their conservation through effective land management. We empirically showed that honeybees are ineffective substitutes for native bees as pollinators of *Diuris* orchids. In *D. brumalis* pollination was higher in the wild habitat where native and alien honeybees co-occurred and was lower in the altered habitats with only introduced honeybees. Pollination was also positively impacted by habitat type and size respectively for *D. brumalis* and *D. magnifica*. Our study provides evidence that biological invasion by *Apis mellifera* can impact orchid pollination and that this effect is exacerbated or even might be triggered by habitat disturbance (altered and fragmented habitats). However, *Apis mellifera* might provide a limited pollinator service for *D. brumalis* and *D. magnifica* where native bees no longer exist, such as disturbed and small fragments of habitat. This indicates that the impact of introduced honeybees on orchid pollination varies with context, necessitating individual evaluations of their effects in each case study. Our findings recommend an accurate and considered management of beekeeping in natural areas and caution against introduction of honeybees to new areas, without carefully determining the minimum ‘safe’ distance of hives to orchid populations and monitoring the number of honeybees relative to native bees in the sites where hives are located. This knowledge is required for ensuring the survival of many orchid species, especially where the habitat is altered and the effect of introduced honeybees on orchid reproductive success is likely to be most severe.

## Methods

### Literature review: incidence of honeybees in pollination of orchids

We searched the global literature to identify and summarise studies in which native and introduced honeybees have been reported as visitors, pollen removers, and depositors in orchid species. In Google Scholar and Web of Science Core Collection we searched the following key words: ‘Apis’, ‘pollinat’, ‘visitor’, ‘introduced bee’, ‘invasive bee’ and ‘honeybee’ and ‘orchid’. The first search was conducted on 1st July 2022 and repeated on 1st March 2023 any paper that mentioned an orchid-honeybee interaction was included. In addition, we included records from available orchid pollination databases^86,89^, books, our photos, and personal observations in which invasive honeybees were reported as a pollen removers of Australian orchid species. During the survey, the introduced honeybee was recorded as a visitor (V, when only observed landing on a flower); pollen depositor (PD, when successfully pollinating the flowers at least once or determined by assessing the configurational features between the flower reproductive structures and bee) or pollen remover (PR, when removing pollinia at least once).

### Study species

*Diuris* (Orchidaceae) comprises approx. 120 species distributed principally in Australia, with centres of diversity in south-western and south-eastern Australia^103^. *Diuris* are terrestrial geophytes, producing a solitary scape per plant yearly; some species within the genus seem capable of clonal reproduction through vegetative propagation of tubers^104^. We selected two allogamous and self-compatible species, *Diuris brumalis* and *D. magnifica*, pollinated via mimicry of co-flowering rewarding legumes by native bees of genus *Trichocolletes*^36,38^. *Apis* was observed to act as a pollen remover of both species (Fig. 2a–e).

Endemic to southwestern Australia, *Diuris brumalis*, is very common in the Darling Range, to the immediate east of Perth, and produces yellow and reddish nectar-less flowers during July and August, with between three and 15 flowers per inflorescence^105^. *Diuris magnifica* is endemic to the Swan Coastal Plain in Western Australia, with its main distribution centred on the Perth metropolitan area^105^ (Fig. 1). Flowering occurs from late winter to early spring, with between three and nine yellow and purple flowers per inflorescence^105^. Given that the species were visited by introduced honeybees and occupied two different habitats, subject to anthropogenic alteration, they were chosen as model species to test for our hypothesis.

### Study sites

We studied 14 populations of *D. brumalis* in the Darling Range, near Perth in Western Australia (Fig. 1). The populations were selected across two different habitat types: Jarrah Forest (hereafter referred to as ‘forest’) dominated by *Eucalyptus marginata* with *Corymbia calophylla* and open Jarrah Forest with *Eucalyptus marginata* and *Allocasuarina fraseriana* highly subjected to fragmentation due to urbanization (hereafter referred to as ‘disturbed woodland’). Populations of *D. magnifica* were distributed across 15 sites in bushland remnants within the metropolitan area of the city of Perth (Fig. 1). Habitat was uniform across populations and characterised by *Banksia* woodland, an ecological community adjacent to the Swan Coastal Plain of Perth with a tree layer of *Banksia* with scattered *Eucalyptus* or *Allocasuarina* species and a diverse understorey including sclerophyllous shrubs, graminoids and forbs. Both the orchid species co-flowered with a range of Faboideae that represent a conspicuous component of the understorey vegetation.

### Orchid pollination success

The proportion of pollen removed (proxy of male fitness) and fruits (proxy of female fitness) come from previously published studies^36,38^ for *D. brumalis* and *D. magnifica* respectively (Datafile S1). Data from two additional populations were included to increase the sample size for *D. magnifica*. For *D. brumalis* the proportion of flowers with pollinia removal and the proportion of pollinated flowers at the end of the flowering period (i.e., the number of flowers found with at least one pollen massula on the stigma) was quantified in 2016 and in 2017, using a 30 × 30 m quadrat centred in each population. As per *D. brumalis*, at the end of flowering period in 2017, the proportion of *D. magnifica* flowers with pollinia removal and the proportion of pollinated flowers was recorded.

### Observational transects on pollinator occurrence

We carried out observations along transects of 100 m length for 10 sites (populations) in September 2016 and 14 sites in September 2017 during *D. brumalis* flowering period. We recorded the occurrence of the native pollinator, *Trichocolletes* spp. (Colletidae) bees, and the introduced honeybee by observing all the flowering species of the understory vegetation along the transect and habitat type. Transects were centred on the same quadrats used to quantify pollination success of *D. brumalis* (see former paragraph). Observations along transects lasted 40 min for both species, spending approximately 3 min per flowering plant. Transects were repeated one week after the initial survey, following the same route. For *D. magnifica* we carried out two observation transects for all the bushland reserves, from 5th to 15th September 2017, by recording the frequency (number of insects) of native *Trichocolletes* spp. bees, and the introduced honeybee during 3 min of observation per flowering plant. Beetles of *Neophyllotocus* sp. (Scarabeideae; Coleoptera) were included too because they have been observed to extract pollinia and deposit it on the stigma of the same orchid flower on two occasions^38^. Sizes of bushland reserves were obtained from Scaccabarozzi et al.^38^. To quantify the effectiveness of pollen transfer, we calculated pollination efficiency (PE) for each population of both species, expressed as a ratio of Fp/Fr where Fp is the number of pollinated flowers and Fr is the number of flowers found with one or both pollinia removed^30,31^. The value of PE potentially ranges between 0 and 1, with 1 representing the maximum and 0 the lowest efficiency.

### Statistical analysis

We analysed the relationship between the proportion of pollinia removed, proportion of fruits, and pollination efficiency with the following independent variables via generalised linear mixed models (GLMs): co-occurrence of honeybees and native bees, lack of co-occurrence (for *D. brumalis*), and abundance of honeybees and abundance of native bees (for *D. magnifica*). Year was included in each model as a fixed factor, while population was included as a random effect to account for repeated measures over time.

We also evaluated the effect of pollinator occurrence and year on (i) the proportion of pollinia removed, (ii) the proportion of fruits on number of flowering plants and (iii) the pollination efficiency in *D. brumalis*. To do so, we employed generalized linear regression models (GLMs) with binomial or quasi-binomial distributions of the response variables, depending on the overdispersion parameter. We first evaluated the role of the factor sampling site on the response variables to avoid possible data dependency. Regression models were evaluated for collinearity among covariates using the VIF criterion (VIF < 3). All the models were subjected to a backward regression approach to remove non-significant variables through the AICc criterion (delta AICc > 2). For *D. magnifica* we wanted to assess the effect of habitat size on orchid pollination success (pollinia removed and fruit set) and pollination efficiency. To do so, we tested the effects of number of plants, native and honeybee abundance, beetle abundance, and remnant size on the same response variables analysed for *D. brumalis*. The statistical analyses followed the same workflow described above. Honeybee and native abundance were not collinear. Furthermore, the relationship between remnant size and native bee abundance was evaluated through a negative binomial GLM to account for the overdispersion of the residuals occurring in the Poisson model. All the analyses were carried out in R ver 4.2.0 (R Core Team 2022) exploiting the following packages “ggplot2”, “plyr”, “MuMIn”, “mass”^106–109^.

### Supplementary Information

Supplementary Information 1.

Supplementary Information 2.

## Data Availability

All data generated or analysed during this study are included in this published article and its Supplementary Information files. Supplementary material associated with this article includes Figure S1, Figure S2, Table S1, Table S2 and Datafile S1. Experimental research and field studies on study plants comply with relevant institutional, national, and international guidelines and legislation. Permission to collect *Diuris brumalis* and *D. magnifica* for identification purposes were obtained and collected specimens were vouchered and identified by the Herbarium of Western Australia, Perth. The material is publicly available at the Herbarium. Vaucher numbers: DS004, DS009, DS010, DS013, DS018.
