# Supplementary material for: Evidence of introduced honeybees (Apis mellifera) as pollen wasters in orchid pollination
Source: Sci Rep. 2024 Jun 18;14:14076. doi: 10.1038/s41598-024-64218-x (PMC11189403; doi:10.1038/s41598-024-64218-x)
Supplement: Supplementary file 2 — Supplementary Information 2. [file 41598_2024_64218_MOESM2_ESM.docx]

**Evidence of introduced honeybees (*Apis mellifera*) as potential pollen wasters in orchid pollination**

Daniela Scaccabarozzi, Lorenzo Guzzetti, Emiliano Pioltelli, Mark Brundrett, Andrea Aromatisi, Giovanni Polverino, Mario-Vallejo-Marin, Salvatore Cozzolino, Zong-Xin Ren

**Fig. S1.** Introduced honeybees (*Apis mellifera*) captured while visiting the following orchid species in southwestern Australia, a: *Caladenia xantha*; b: *Eriochilus dilatatus;* c: *Prasophyllum elatum* and in eastern Australia, d: *Dendrobium kingianum* carrying three orchid pollinia; e: *Caladenia flava*; f: *Prasophyllum elatum;* g: *Prasophyllum* sp (approaching the flower with pollinia on mouthpart). Photo credits: Mark Brundrett (A, B, C. E), and Rudie Kruiter (D, F, G).


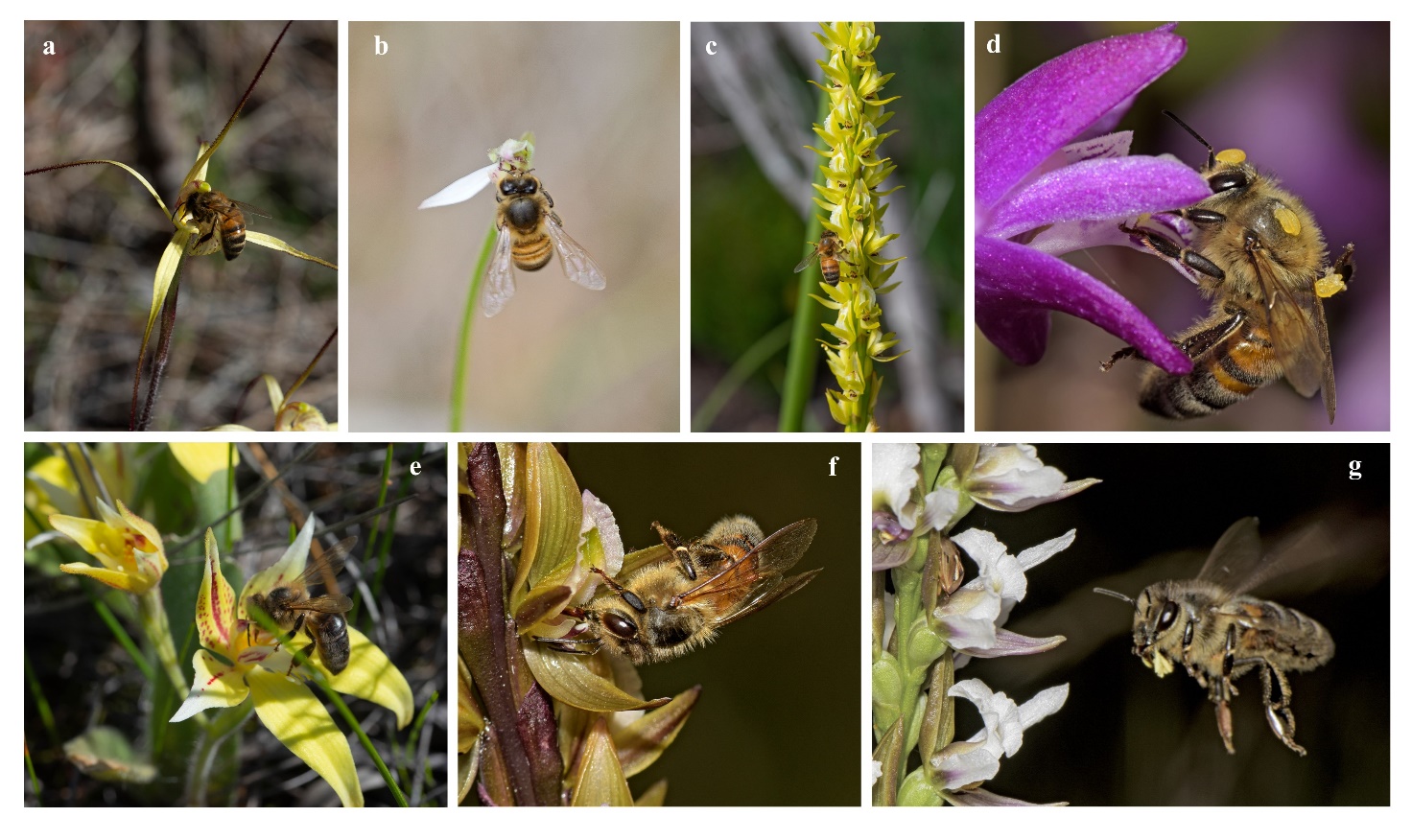


**Fig. S2.** Records (%) of native *Apis* bees and not-native honeybee (*Apis mellifera*) pollinating orchid species from our literature survey (Table 1) a: proportion of native honeybees involved in orchid pollination among continents; b: proportion of introduced honeybees according to pollination effectiveness; c: proportion of introduced honeybees per orchid subfamilies.


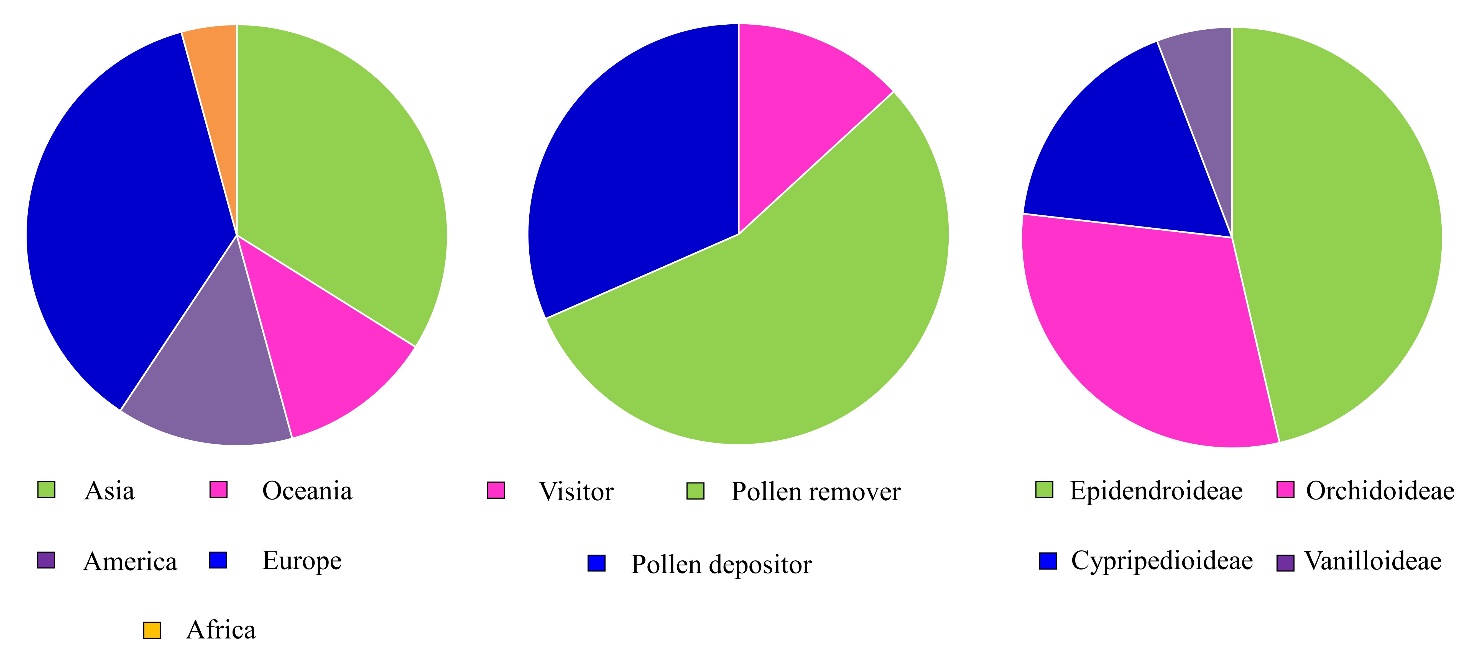


**Table S1.** Literature survey presenting the incidence of *Apis* bees as a native species in orchid pollination across continents.

| **Continent** | **Country** | **Subfamily** | **Plant species** | **Native or alien plant species** | **Native bee or other native pollinators** | **Literature source** |
| --- | --- | --- | --- | --- | --- | --- |
| Europe | Israel | Orchidoideae | *Anacamptis collina* | native | *Apis mellifera* | Dafni and Ivry 1979 |
| Europe | Middle East, Crimea | Orchidoideae | *Anacamptis coriophora* | native | Various Coleoptera, Hemiptera, Diptera, Lepidoptera, Vespidae and bees including *Apis mellifera, Bombus argillaceus, B. humilis, B. pascorum, B. praetorum, B. subterraneus and many others* | Dafni and Ivri 1979; Cozzolino et al. 2001; Joffard et al. 2019 |
| Europe | Israel | Orchidoideae | *Anacamptis coriophora subsp. fragrans* | native | *Vespula vulgaris, Xylocopa iris, Zygaena grasilini* (Lepidoptera)*, Apis mellifera* | Dafni and Ivry 1979 |
| Europe | Austria and South of Italy | Orchidoideae | *Anacamptis morio* | native | *Bombus sp., Apis mellifera, Andreana* sp., *Eucera* sp. and other bees | Vöth 1987; Cozzolino et al. 2005 |
| Europe | Greece | Orchidoideae | *Anacamptis papilionacea* | native | *Apis mellifera, Nomada* sp*., Osmia* sp*., Eucera* sp. | Vöth 1989 |
| Europe | Austria | Orchidoideae | *Anacamptis pyramidalis* | native | *Apis mellifera, Zygaena purpuralis* | Vöth 1999 |
| Asia | China | Epidendroideae | *Bulbophyllum ambrosia* | native | *Apis cerana* | Chen and Gao 2011 |
| Asia | Japan | Epidendroideae | *Calanthe discolor* | native | *Apis cerana japonica, Eucera nipponensis, Osmia cornifrons* | Suetsugu and Fukushima 2014 |
| Asia | China | Epidendroideae | *Changnienia amoena* | native | *Bombus tritasciatus,* likely *Apis cerana* | Sun et al. 2003 |
| Asia | China (Yunnan) | Epidendroideae | *Cleisostoma linearilobatum* | native | *Amegilla ynnanensis, Apis sp., Megachile dimidiate, Vespa* sp*., Eumenes 2 spp., Prionyx sp.* | Zhou et al. 2016 |
| Asia | India Nagaland | Epidendroideae | *Coelogyne corymbosa* | native | *Apis indica* | Chaturvedi 2009 |
| Asia | SE Asia | Epidendroideae | *Coelogyne fimbriata* | native | *Apis cerana, Vespula sp., Provespa nocturna* | Cheng et al. 2009; Nakase and Kato 2012; Liu et al. 2013 |
| Asia | Nepal | Epidendroideae | *Coelogyne flaccida* | native | *Apis cerana* | Subedi et al. 2011 |
| Asia | Nepal | Epidendroideae | *Coelogyne nitida* | native | *Apis cerana* | Subedi et al. 2011 |
| Asia | South Eeast Asia | Epidendroideae | *Coelogyne pulverula* | native | *Apis cerana* | Ong and Chen 2019 |
| Asia | China (Yunnan) | Epidendroideae | *Coelogyne rigida* | native | *Apis cerana, Aethopyga gouldiae, Vespula* sp. | Liu et al. 2013 |
| Asia | South Eeast Asia | Epidendroideae | *Coelogyne viscosa* | native | *Apis cerana* | Wang 2009 |
| Asia | India | Epidendroideae | *Cymbidium aloifolium* | native | *Apis cerana indica* | Adit et al. 2022 |
| Asia | Malaysia | Epidendroideae | *Cymbidium atropurpureum* | native | *Apis* | Koeniger et al. 2016 |
| Asia | Japan | Epidendroideae | *Cymbidium dayanum* | native | *Apis cerana japonica* | Matsuda and Sugiura 2019 |
| Asia | Nepal, Sikkim, Bhutan, and North Thailand | Epidendroideae | *Cymbidium devonianum* | native | *Apis cerana japonica* | Sugahara 2006 |
| Asia | Borneo, Malesia | Epidendroideae | *Cymbidium finlaysonianum* | native | *Apis cerana* | Chan et al. 1994; Ong 2010 |
| Asia | Japan | Epidendroideae | *Cymbidium floribundum* | native | *Apis cerana japonica* | Sugahara et al. 2013; Sugahara et al. 2010; Sasagawa et al. 2005 |
| Asia | China | Epidendroideae | *Cymbidium goeringii* | native | *Apis cerana* | Yu et al. 2008 |
| Asia | Japan | Epidendroideae | *Cymbidium goeringii* | native | *Apis cerana japonica* | Tsuji and Kato 2010 |
| Asia | Japan | Epidendroideae | *Cymbidium kanran* | native | *Apis cerana japonica* | Tsuji and Kato 2010 |
| Asia | China | Epidendroideae | *Cymbidium lancifolium* | native | *Apis cerana* | Cheng et al. 2007 |
| Asia | Japan | Epidendroideae | *Cymbidium lancifolium* | native | likely *Apis cerana cerana* | Suetsugu 2015 |
| **Continent** | **Country** | **Subfamily** | **Plant species** | **Native or alien plant species** | **Native bee or other native pollinators** | **Literature source** |
| Asia | Japan | Epidendroideae | *Cymbidium macrorhizon* | native | *Apis cerana cerana* | Suetsugu 2015 |
| Asia | Japan | Epidendroideae | *Cymbidium pumilum* | alien | *Apis cerana japonica* | Sasaki et al. 1991 |
| Asia | China | Epidendroideae | *Cymbidium qiubeiense* | native | *Apis cerana cerana* | Hu et al. 2018 |
| Asia | Myanmar | Epidendroideae | *Cymbidium suavissimum* | native | *Apis cerana japonica* | Sugahara 2006 |
| Asia | China | Epidendroideae | *Cymbidrium faberi* | native | *Apis cerana* | Yang 2017 |
| Europe | Poland | Orchidoideae | *Dactylorhiza majalis* | native | *Apis mellifera* | [Ostrowiecka et al. 2019](https://www.webofscience.com/wos/author/record/12393607) |
| Europe | France | Orchidoideae | *Dactylorhiza majalis* | native | *Bombus* sp.*, Apis mellifera* | Berger 2003 |
| Europe | EU countries | Orchidoideae | *Dactylorhiza ochroleuca* | native | *Apis mellifera* | Claessens and Kleynen 2011 |
| Europe | Sweden | Orchidoideae | *Dactylorhiza sambucina* | native | *Bombus* sp.*, Osmia bicolor, Apis mellifera* | Nilsson 1980 |
| Europe | EU countries | Orchidoideae | *Dactylorhiza sphagnicola* | native | *Apis mellifera* | Claessens and Kleynen 2013a |
| Europe | Poland and England | Orchidoideae | *Dactylorhiza spp. (D. incarnata, D. fuchsii* and *D. majalis)* | native | *Apis mellifera* | Wroblewska et al. 2019; Ostrowiecka et al. 2019; Dafni and Woodell 1986 |
| Europe | EU countries | Orchidoideae | *Dactylorhiza sudetica* | native | *Leptura fulva, Lepturobosca virens* (Coleoptera)*, Apis mellifera, Bombus lapidarius* | Claessens and Kleynen 2011 |
| Europe | EU countries | Orchidoideae | *Dactylorhiza viridis* | native | Various Coleoptera, *Dolerus gonager, Tenthredopsis tarsata, T. sp. (Tenthredinidae sawfly), Mesoleptus transversator (Ichneumonidae), Apis mellifera (Apidae), Coptoformica exsecta (Formicidae)* | Hagerup 1952; Claessens and Seifert 2017; Joffard et al. 2019 |
| Asia | Singapore | Epidendroideae | *Dendrobium anosmum* | native | *Apis dorsata, A. indica* | van der Pijl and Dodson 1966 |
| Europe | EU countries | Epidendroideae | *Epipactis atrorubens* | native | *Apis mellifera, Bombus hypnorum, B. jonellus, B. lapidarius, B. lucorum, B. mastrucatus, B. pascuorum, B. pratoru, Dufourea dentiventris, Osmia caerulescens; Delta unguiculata, Dolichovespula saxonica, D. sylvestris, Odynerus spinipes, Vespa diabolica* | Claessen and Kleynen 2011; Brys and Jacquemyn 2015; Godfrey 1933; Claessens and Kleynen 2011 |
| Europe | Sweden | Epidendroideae | *Epipactis palustris* | native | *Apis mellifera, Andrena* sp.*, Lassioglossum* sp.*, Bombus* and other insects | Nilsson 1978 |
| Europe | Poland and Czech | Epidendroideae | *Epipogium aphyllum* | native | *Apis mellifera* | Jakubska-Busse et al. 2014 |
| Europe | Czech Republic & Poland | Epidendroideae | *Epipogium aphyllum* | native | *Apis mellifera, Bombus* sp. | Jakubska-Busse et al. 2014 |
| Asia | China | Epidendroideae | *Epipogium roseum* | native | *Apis cerana cerana* | Zhou et al. 2012 |
| Asia | China | Epidendroideae | *Eria coronaria* | native | *Apis cerana* | Shangguan et al. 2008 |
| Asia | SE Asia, Malaysia | Epidendroideae | *Eria densa* | native | *Apis cerana* | Ong 2010 |
| Asia | SE Asia | Epidendroideae | *Eria floribunda* | native | *Apis cerana* | Carr 1928 |
| Asia | China | Orchidoideae | *Goodyera foliosa* | native | *Apis cerana* | Zha et al. 2016; Liu et al. 2020 |
| Europe | Germany | Orchidoideae | *Goodyera repens* | native | *Bombus* sp*., Lassioglossum* sp.*, Apis mellifera* | Vöth 1999; Classens and Kleynen 2013b |
| Asia | Singapore | Epidendroideae | *Grammatophyllum speciosum* | native | *Apis dorsata, Augochlora pura, Vespa cincta* (Vespidae) | Ridley 1905; van der Pijl and Dodson 1966; van der Cingel 2001 |
| Europe | France | Orchidoideae | *Gymnadenia corneliana* | native | *Apis mellifera, Colias phicomone* (Lepidoptera)*, Erebia alberganus* (Lepidoptera) | Berger 2009 |
| Europe | EU countries | Orchidoideae | *Gymnadenia lithopolitanica* | native | *Apis mellifera* | Vöth 2000; Claessens and Kleynen 2001 |
| Europe | EU countries | Orchidoideae | *Gymnadenia nigra* | native | Various Diptera, Lepidoptera an*d Apis mellifera* | Kaiser 1993; Müller 1874; Müller 1881; Godfrey 1933 |
| Europe | Austria | Orchidoideae | *Himantoglossum adriaticum* | native | *Apis mellifera, Colletes similis* | Vöth 1999 |
| Europe | Hungary | Orchidoideae | *Himantoglossum adriaticum* | native | *Apis mellifera* (Apiary) | Biro et al. 2014 |
| **Continent** | **Country** | **Subfamily** | **Plant species** | **Native or alien plant species** | **Native bee or other native pollinators** | **Literature source** |
| Europe | Eu countries | Orchidoideae | *Himantoglossum hircinum* | native | *Oedemera nobilis (Coleoptera), Andrena bicolor, A. carbonaria, A. cf. carantonica, A. cineararia, A. fulva, A. haeamorrhoa, A. jacobi, A. nigroaenea (Andrenidae); Apis mellifera, Bombus lucorum, B. terrestris, Colletes cunicularius, Megachile martima, Odynerus parietum* (Vespidae). | Carey and Farrell 2002; Claessens and Kleynen 2011, 2016; Berger 2007; Teschner 1980 |
| Europe | Eu countries | Orchidoideae | *Himantoglossum jankae* | native | *Apis mellifera, Eucera tricincta, E. nigra, Anthidium cingulatum, A. loti, Coelioxys caudata, Megachile ericetorum, M. parietina, M. lefebvrei, M. ericetorum, M. pilicrus* | Claessens and Kleynen 2016; Ivanov et al. 2011 |
| Europe | Sweden | Orchidoideae | *Neotinea ovata* | native | *Apis mellifera* and a broad range of insects | Nilsson 1981 |
| Europe | Middle East, Crimea | Orchidoideae | Neotinea tridentata | native | Various Coleoptera, Diptera and bees including *Andrena cineraria, Apis mellifera, Bombus hortorum, B. terrestris, Nomada fucata, Halictus patellatus, Lasioglossum sp., Osmia aurulenta, O. bicolor* | Ivanov et al. 2009; Cozzolino et al. 2001, 2005; Claessens and Kleynen 2011; Voth 1999 |
| Europe | EU countries | Orchidoideae | *Orchid simia* | native | Various Coleoptera, Lepidoptera and bees including *Apis mellifera, Eucera longicornis* | Schatz 2006; Fantinato et al. 2017; Joffard et al. 2019 |
| Europe | EU countries | Orchidoideae | *Orchids pauciflora* | native | Various Lepidoptera and bees including *Anthophora mucida, A. plumipes, Apis mellifera, Bombus humilis, B. terrestris, B. lapidarius* | Ivanov et al. 2009; Fantinato et al. 2017; Claessens and Kleynen 2011; Joffard et al. 2019 |
| Europe | Greek | Orchidoideae | *Orchis boryi* | native | *Apis mellifera* | Gumbert and Kunze 2001 |
| Europe | Middle East | Orchidoideae | *Orchis collina* | native | *Apis mellifera* | Dafni and Ivri 1979 |
| Europe | Greece | Orchidoideae | *Orchis italica* | native | *Apis mellifera, Chelostoma transversum, Anthidium septemdentatum* | Vöth 1998 |
| Europe | EU contries | Orchidoideae | *Orchis mascula* | native | Various Coleoptera, Lepidoptera, Diptera, and bees including *Andrena bicolor, A. haemorrhoa, A. helvola, A. nigroaenea, A. nitida, Apis mellifera, Bombus barbutellus* | Cozzolino et al. 2005; Barile et al. 2006; Nilsson 1983; Bellusci et al. 2010 |
| Europe | Austria | Orchidoideae | *Orchis militaris* | native | *Apis mellifera, Andrea* sp*., Halictus* sp*., Osmia* sp*., Tropinota hirta* (Coleoptera) | Vöth 1999 |
| Europe/Africa | EU countries, North Africa | Orchidoideae | *Orchis ovalis* | native | *Apis mellifera, Bombus pascuorum* | Claeussens and Kleynen 2011 |
| Europe | EU countries | Orchidoideae | *Orchis punctulata* | native | Apis; Halictidae | Tsiftsis and Djordjevic 2018 |
| Europe | France | Orchidoideae | *Orchis purpurea* | native | *Apis mellifera* | Berger 2004 |
| Europe | EU countries | Orchidoideae | *Spiranthes aestivalis* | native | *Apis mellifera, Bombus pascuorum, B. pratorum, B. terrestris, Halictus* sp.*, Lasioglossum* sp. | Claessens and Kleynen 2011; Joffard et al. 2019 |
| America | Colorado, Utah | Orchidoideae | *Spiranthes diluvialis* | native | *Anthophora terminalis, Apis mellifera, Bombis appositus, B. bifarius, B. fervidus, B. griseocollis, B. morissoni, B. occidentalis, B. rufocinctus,* Tiphiidae wasps | Sipes and Tepedino 1995; Pierson et al. 2001 |
| Europe | Ireland | Orchidoideae | *Spiranthes romanzoffiana* | native | *Apis mellifera* | Duffy and Stout 2008 |
| Asia | China | Orchidoideae | *Spiranthes sinensis* | native | *Apis cerana, Bombus* sp., *Ceratina*, Halictidae | Tao et al. 2018 |
| Europe | Greek | Orchidoideae | *Spiranthes spiralis* | native | *Apis mellifera* | Petanidou et al. 2013 |
| Europe | NA | Orchidoideae | *Spiranthes spiralis* | native | *Apis mellifera* | Reinhard et al. 1991 |
| Europe | Austria | Orchidoideae | *Traunsteinera globosa* | native | *Bombus* sp.*, Lassioglossum* sp.*, Apis mellifera* and other insects | Vöth 1994 |

**Table S2.** Statistical parameters relative to the employed model using continuous predictor variables for the study species *Diuris magnifica* (Orchidaceae).

| **Response variable** | **Model covariates** | **Final model covariates** | **B_i_** | χ2;df | **p-value** |
| --- | --- | --- | --- | --- | --- |
| Male fitness | Native bees’ abundance | Native bees’ abundance | 0.05278 | 19.572; 1 | <0.001 |
|  | Number of plants |  |  |  |  |
|  | Honeybees’ abundance |  |  |  |  |
|  | Beetles’ abundance |  |  |  |  |
|  | Remnant size |  |  |  |  |
| Female fitness | Native bees’ abundance | Native bees’ abundance | 0.06411 | 5.1371; 1 | 0.023 |
|  | Number of plants |  |  |  |  |
|  | Honeybees’ abundance |  |  |  |  |
|  | Beetle abundance |  |  |  |  |
|  | Remnant size |  |  |  |  |
| Pollination efficiency | Native bees’ abundance | I(Honeybees’ abundance) ^2 | -0.014423 | 7.2195; 1 | 0.007 |
|  | Number of plants | Log(Remnant size) | 0.551009 | 6.7399; 1 | 0.009 |
|  | Honeybee abundance |  |  |  |  |
|  | Beetle abundance |  |  |  |  |
|  | Remnant size |  |  |  |  |

**Additional References from Table S1.**

Adit A, Koul M, Choudhary AK, Tandon R (2022) Interaction between *Cymbidium aloifolium* and *Apis cerana*: Incidence of an outlier in modular pollination network of oil flowers. Ecol Evol 12:e8697. <https://doi.org/10.1002/ece3.8697>

Backhouse G, Bates RJ, Brown AP, Copeland LM (2019) A Checklist of the Orchids of Australia Including its Island Territories. Self-published, Melbourne. ISBN 978-0-9946489-3-8.

Barile, MC, Cozzolino S, De Castro O, Lucchese F, Zilli A (2006) New insights into pollination of Mediterranean terrestrial orchids by butterflies and moths. Jour Eur Orch 38: 449-460.

Bellusci F, Musacchio A, Stabile R, Pellegrino G (2010) Differences in pollen viability in relation to different deceptive pollination strategies in Mediterranean orchids. Ann Bot doi:10.1093/aob/mcq164

Berger L. (2003) Observations sur le comportement de quelques pollinisateurs d’orchidées (2e partie) L’Orchidophile 159: 277-290.

Berger L (2009) La pollinisation du genre *Nigritella* (L.C.M. Richard). Jour Eur Orch 41:411-432.

Berger L (2007) Une punaise sur l’orchis punaise. Bull. SFO-Rhône-Alpes 13: 144-24.

Bíró E, Bodis J, Nagy T, Toekoelyi J, Molnar VA (2015). Honeybee (*Apis mellifera*) mediated increased reproductive success of a rare deceptive orchid. Appl Ecol Environ Res 13:181-192.

Brys R, Jacquemyn H (2015) Severe outbreeding and inbreeding depression maintain mating system differentiation in *Epipactis* (Orchidaceae). J Evol Biol doi:10.1111/jeb.12787

Burton K (2009) Mu-Mln: Multi-model inference. R package, Version 0.12.2/r18. <https://mumin.r-forge.r-project.org/>

Carey PD, Farrell (2002) Biological Flora of the British Islaes. *Himantoglossum hircinum* (L.) Sprengel. J Ecol 90: 206-218.

Carr CE (1928) Orchid pollination notes. JMBRAS 6: 49-72.

Chaturvedi SK (2009) Mechanism of pollination in *Coelogyne corymbosa* Lindl. (Orchidaceae). Int J Plant Reprod Biol 1: 133-135.

Chen L, Gao J (2011) Reproductive ecology of *Bulbophyllum ambrosia* (Orchidaceae). Acta Pharmacol Sin 35:1202-1208. <https://www.plant-ecology.com/EN/10.3724/SP.J.1258.2011.01202>

Chan CL, Lamb A, Shim PS, Wood JJ (1994) Orchids of Borneo, volume 1: introduction and a selection of species. Sabah Society Kota Kinabalu, Kew, Royal Botanic Gardens, Kew, London.

Cheng J, Shi J, Shangguan F-Z, Dafni A, Deng Z-H, Lou Y-B (2009) The pollination of a self-incompatible, food-mimic orchid, *Coelogyne fimbriata* (Orchidaceae), by female Vespula wasps. Ann Bot 104: 565-571. 10.1093/aob/mcp029

Chung MY, Chung MG (2005) Pollination biology and breeding systems in the terrestrial orchid *Bletilla striata*. Plant Syst Evol. 252:1-9. <https://doi.org/10.1007/s00606-004-0256-6>

Claessens J, Kleynen J (2011) The flower of the European orchid: form and function. Claessens & Kleynen, Netherlands

Claessens J, Kleynen J. (2013)a The pollination of European orchids. Part 1: Introduction and the genera *Orchis* and *Dactylorhiza*. JHOS 10: 83-89

Claessens J, Kleynen J (2013)b Honigbienen (*Apis mellifera*) als Bestäuber von *Goodyera repens*. Jour Eur Orch 45:133-138.

Claessens J, Kleynen J (2016) The pollination of European orchids. Part 5: *Himantoglossum* and *Anacamptis.* JHOS 13: 114-123.

Claessens J, Seifert B (2017) Significant ant pollination in two orchid species in the Alps as adaptation to the climate of the alpine zone? Tuexenia 37: 363-374.

Cozzolino S, Aceto S, Caputo P, Widmer A, Dafni A. (2001) Speciation processes in Eastern Mediterranean *Orchis* s.l. species: molecular evidence and the role of pollination biology. Isr J Plant Sci 49:91-103.

Dafni A, Yariv I (1979) Pollination ecology of, and hybridization between *Orchis coriophora* L. and *O. collina* Sol. Ex Russ. (Orchidaceae) in Israel. New Phytol 83:181-187.

Dafni A, Woodell SRJ (1986) Stigmatic Exudate and the Pollination of *Dactylorhiza fuchsii* (Druce) Soo. Flora 178:343-350. <https://doi.org/10.1016/S0367-2530(17)31521-9>

Dixon KW, Buirchell BJ, Collins MT (1989) Orchids of Western Australia: Cultivation and Natural History, 2^nd^ ed. Western Australian Native Orchid Study and Conservation Group, Victoria Park, Perth.

Diller C, Castañeda-Zárate M, Johnson SD (2022) Why honeybees are poor pollinators of a mass-flowering plant: experimental support for the low pollen quality hypothesis. Am J Bot 109:1305-1312. https://doi.org/10.1002/ajb2.16036

Dodson CH (1966) Ethology of some bees of the tribe Euglossini (Hymenoptera: Apidae). J Kans Entomol Soc 39:607-629. <https://www.jstor.org/stable/25083566>

Duffy KJ, Stout JC (2008) The effects of plant density and nectar reward on bee visitation to the endangered orchid *Spiranthes romanzoffiana*. Acta Oecol 34:131-138. <https://doi.org/10.1016/j.actao.2008.04.007>

Fantinato E, Del Vecchio S, Baltieri M, Fabris B, Buffa G (2017) Are food-deceptive orchid species really functionally specialized for pollinators? Ecol Res 32: 951-959. <https://doi.org/10.1007/s11284-017-1501-0>

Godfrey MJ (1933) Monograph and iconograph of native British orchids. Cambridge University Press.

Government of Western Australia (2000) Bush forever, policies, principles and processes, Vol. 1. Western Australian Planning Commission, Perth.

Koeniger G, Koeniger N, O’byrne P, Lamb L, Phiancharoen M, Lim H, Tihoi S, Bosuang S (2017): Scientific note on *Apis koschevnikovi* chewing petals of an orchid flower (*Dipodium* species O’Byrne KIP1272) in Borneo. Apidologie (Celle) 48:259-261. <https://doi.org/10.1007/s13592-016-0470-6>

Gumbert A, Kunze J (2001) Colour similarity to rewarding model plants affects pollination in a food deceptive orchid, *Orchis boryi*. Biol J Linn 72:419-433. <https://doi.org/10.1111/j.1095-8312.2001.tb01328.x>

Hagerup O (1952) Bud autogamy in some northern orchids. Phytomorphology 2:51-60.

Henneresse T, Tyteca D (2016) Insect Visitors and Potential Pollinators of *Orchis militaris* (Orchidaceae) in Southern Belgium. J Insect Sci 16:104. <https://doi.org/10.1093/jisesa/iew088>

Hoffman N, Brown A (2011) Orchids of south-west Australia, 3^rd^ ed., University of Western Australia, Perth.

Hu S, Xin R, Guo H, Wang X, Zhang Z, Cheng J (2018) Accuracy detection of predicting pollinator from pollination syndromes: taking *Cymbidium qiubeiense* as an example. J Beijing for Univ 40:101-110.

Ivanov SP, Kholodov VV, Fateryga AV (2009) Orchids of Crimea: composition of pollinators, variety of systems and methods of pollination and their efficiency. Scientific notes of the VI. Cernadsky Crimean Federal University. Biology-Chemistry 22: 24-34. [in Russian]

Ivanov SP, Fateryga AV, Kholodov VV 2011 Pollination ecology of Lizard Orchid (*Himantoglossum caprinum*) in Crimea. In Shamrov II (ed.). Okhrana I kultivirovanijy orkhidey [Protection and cultivation of orchids]. Moscow: KMK Scientific Press, p. 187-194. [in Russian]

Jakubska-Busse A, Jasicka-Misiak I, Poliwoda A, Święczkowska E, Kafarski P (2014) The chemical composition of the floral extract of *Epipogium aphyllum* Sw. (Orchidaceae): a clue for their pollination biology. Arch Biol Sci 66:989-998. 10.2298/ABS1403989B

Jin C, Shiyong L, Rong H, Xinlian W, Yibo L (2007) Food-deceptive pollination in *Cymbidium lancifolium* (Orchidaceae) in Guangxi, China. Biodiv Sci 15:608-617. <https://www.biodiversity-science.net/EN/10.1360/biodiv.070091>

Kaiser R (1993) The scent of orchids: olfactory and chemical investigations. Ediciones Roche, Basel.

Li P, Huang BQ, Pemberton RW, Luo YB, Cheng J (2011) Floral display influences male and female reproductive success of the deceptive orchid *Phaius delavayi*. Plant Syst Evol 296:21-27. <https://www.jstor.org/stable/43558601>

Liu ZJ, Chen LJ, Liu KW, Li LQ, Rao WH, Zhang Y-T, Tang G-D, Huang L-Q (2013) Adding perches for cross-pollination ensures the reproduction of a self-incompatible orchid. 8:e53695 <https://doi.org/10.1371/journal.pone.0053695>

Liu N, Xiao H, Chen X, Chi Y, Luo H, Xiong D, Yang B (2020) Nectar secretion characteristics and their effects on insect pollination of *Goodyera foliosa*, a nectar rewarding orchid. J Trop Subtrop Bot 28:265-270. <https://doi.org/10.1007/s00606-011-0473-8>

Luer CA (1972) The native orchids of Florida. New York Botanical Garden, New York.

Lussu M, De Agostini A, Cogoni A, Marignani M, Cortis P (2019) Does size really matter? A comparative study on floral traits in orchids with two different pollination strategies. Plant Biol J 21:961-966. <https://doi.org/10.1111/plb.12993>

Matsuda Y, Sugiura N (2019) Specialized pollination by honeybees in *Cymbidium dayanum*, a fall-winter flowering orchid. Plant Species Biol 34:19-26. <https://doi.org/10.1111/1442-1984.12231>

Müller H (1874) Fertilisation of flowers by insects IX. Alpine orchids adapted to cross-fertilisation by butterflies. Nature 11: 169-171.

Müller H (1881) Alpenblumen, ihre Befruchtung durch Insekten und ihre Anpassungen an dieselben. Leipzig.

NakaseY, Kato M (2012) A nocturnal Provespa wasp species as the probable pollinator of epiphytic orchid *Coelogyne fimbriata*. Entomol Sci 15: 253-256.

Nilsson LA (1978) Pollination ecology of *Epipactis palustris* (Orchidaceae). Bot not 131:255– 368.

Nilsson LA (1980) The pollination ecology of *Dactylorhiza sambucina* (Orchidaceae). Bot not 133:367– 385.

Nilsson LA (1983) Anthecology of *Orchis mascula* (Orchidaceae). Nord J Bot 3: 157-179.

Ong PT (2010) Notes on bee pollination in three peninsular Malaysian orchids. Malayan Orchid Review 44:73–75.

Ong PT, Chen C-M (2019) Notes on the pollination of *Coelogyne pulverula* Teijsm. & Binn. in Peninsular Malaysia. Malayan Orchid Review 53: 77-79.

Ostrowiecka B, Talalaj I, Brzosko E, Jermakowicz E, Mirski P, Kostro-Ambroziak A, Mielczarek L, Lason A, Kupryjanowicz J, Kotowicz J, Wroblewska A (2019) Pollinators and visitors of the generalized food-deceptive orchid *Dactylorhiza majalis* in North-Eastern Poland. Biologia 74:1247-1257. <https://doi.org/10.2478/s11756-019-00285-0>

Petanidou T, Duffy KJ, Karatza A, Kantsa A (2013) Reduced fecundity in large populations of a Mediterranean orchid - Evidence for pollinator limitation. Basic Appl Ecol 14:36-43. <https://doi.org/10.1016/j.baae.2012.11.007>

Pierson K, Tepedino VJ, Sipes S, Kuta K (2001) Pollination ecology of the rare orchid, Spiranthes diluvialis: implications for conservation. pp. 153-164. In Maschinski J, Holter L (eds.) Soouthwestern rare and endangered plants: Proceedingsof the Third Conference. US Department of Agriculture, Forest Service, Rocky Mountain Research Station, Fort Collings, Colorado.

R Core Team (2022) R: A language and environment for statistical computing. R Foundation for Statistical Computing, Vienna, Austria. <https://www.R-project.org>

Reinhard HR, Gölz P, Peter R, Wildermuth H (1991) Die Orchideen der Schweiz und angrenzender Gebiete. – Fotorotar, Egg 348 pp.

Ridley HN (1905) On the fertilization of *Grammatophyllum*. JSBRAS 44:228-229.

Sasagawa H, Kadowaki T, Matsuyama S (2005) Honeybee communication system and pollination tactics of an oriental orchid that secretes honeybee semiochemicals. Zool Sci 22: 1513-1513.

Sasaki M, Ono M, Asada S, Yoshida T (1991) Oriential orchid (*Cymbidium pumilum*) attracts drones of the Japanese honeybee (*Apis cerana japonica*) as pollinators Experientia 47:1229-1231. <https://doi.org/10.1007/BF01918392>

Schatz B (2006) Fine scale distribution of pollinator explains the occurrence of the natural orchid hybrid x *Orchis bergonii*. Ecoscience 13:118-118.

Schweiger O, Biesmeijer JC, Bommarco R, Hickler T, Hulme PE, Klotz S, Kühn I, Moora M, Nielsen A, Ohlemüller R, Petanidou T, Potts SG, Pyšek P, Stout JC, Sykes MT, Tscheulin T, Vilà M, Walther GR, Westphal C, Winter M, Zobel M, Settele J (2010) Multiple stressors on biotic interactions: how climate change and alien species interact to affect pollination. Biol Rev 85:777-795. <https://doi.org/10.1111/j.1469-185X.2010.00125>

Shangguan F, Cheng J, Yuanxin X, Yibo L (2008) Deceptive pollination of an autumn flowering orchid *Eria coronaria*

(Orchidaceae). Biodiv Sci 16:477-483. <https://doi.org/10.3724/SP.J.1003.2008.08096>

Sipes SD, Tepedino VJ (1995) Reproductive biology of the rare orchid, *Spiranthes diluvialis*: breeding system, pollination, and implications for conservation. Biol. Conserv 9: 929-938. <http://www.jstor.org/stable/2387001>.

Sugahara M (2006) *Cymbidium devonianum* and *Cymbidium suavissimum* as well as *Cymbidium floribundum* attracts Japanese honeybees (*Apis cerana japonica*). Zool Sci 23 p.1225.

Subedi A, Chaudhary RP, van Achterberg C, Heijerman T, Lens F, van Dooren TJM, Gravendeel B (2011) Pollination and protection against herbivory of Nepalese Coelogyninae (Orchidaceae). Am J Bot 98*:*1095-1103. <https://doi.org/10.3732/ajb.1000306>

Suetsugu K (2015) Autonomous self-pollination and insect visitors in partially and fully mycoheterotrophic species of Cymbidium (Orchidaceae). J Plant Res 128:115-125.

Suetsugu K, Fukushima S (2014) Bee pollination of the endangered orchid *Calanthe discolor* through a generalized food-deceptive system. Plant Syst Evol 300:453-459. <https://doi.org/10.1007/s00606-013-0894-7>

Sugahara M, Izutsu K, Nishimura Y, Sakamoto F (2013) Oriental Orchid (*Cymbidium floribundum*) Attracts the Japanese Honeybee (*Apis cerana japonica*) with a Mixture of 3-hydroxyoctanoic Acid and 10-hydroxy-(E)-2-decenoic Acid. Zool Sci 30:99-104. <https://doi.org/10.2108/zsj.30.99>

Sugahara M, Minamoto T, Fuchikawa T, Michinomae M, Shimizu I (2010) *Apis cerana japonica* Discriminates between Floral Color Phases of the Oriental Orchid, *Cymbidium floribundum*. Zool Sci 27:901-906. <https://doi.org/10.2108/zsj.27.901>

Sun HQ, Luo YB, Ge S (2003) A preliminary study on pollination biology of an endangered orchid, *Changnienia amoena*, in Shennongjia. J Integr Plant Biol 45:1019-1023. <https://www.jipb.net/EN/Y2003/V45/I9/1019>

Tao ZB, Ren ZX, Bernhardt P, Liang H, Li HD, Zhao YH, Wang H, Li DZ. 2018. Does reproductive isolation reflect the segregation of color forms in *Spiranthes sinensis* (Pers.) Ames complex (Orchidaceae) in the Chinese Himalayas? Ecol Evol 8:5455-5469. <https://doi.org/10.1002/ece3.4067>

Teschner W (1980) Sippendifferenzierung und Bestäubung bei Himantoglossum Koch. Jahresber. Naturwiss. Ver. Wuppertal 33: 104-115.

Tsiftsis S, Djordjevic V (2018) Habitat effects and differences in the reproductive success of *Orchis punctulata* and *Orchis purpurea* (Orchidaceae). Turk J Bot 42: doi: 10.3906/bot-1711-22

Tsuji K, Kato M (2010) Odor-guided bee pollinators of two endangered winter/early spring blooming orchids, *Cymbidium kanran* and *Cymbidium goeringii*, in Japan. Plant Species Biol 25:249-253. <https://doi.org/10.1111/j.1442-1984.2010.00294.x>

Venables WN, Ripley BD (2022) Modern Applied Statistics with S. 4^th^ ed., Springer, New York.

Vöth W (1982) Die ‘ausgeborgten’ Bestäuber von *Orchis pallens* L. Die Orchidee 33:196-203.

Vöth W (1987) Bestäubungsbiologische Beobachtungen an *Orchis militaris* L. Die. Orchidee 38:77– 84.

Vöth W (1998) *Orchis italica* und die angetroffenen Insekten. Die Orchidee 49:219-221.

Vöth W (1989) Werden *Ophrys aesculapii* RENZ von *Andrena muscaria* WARNCKE (Andrenidae) bestäubt? Mitt. Bl. Arbeitskr. Heim Orch Baden-Württ 21:133-142.

Vöth W (1994) Bestäuber und Besucher der Blüten von *Traunsteinera globosa* (L.) Rchb., Orchidaceae, in Niederösterreich. – Linzer Biologische Beitraege 26:133-148.

Vöth W (1999) Lebensgeschichte und Bestäuber der Orchideen am Beispiel von Niederösterreich. Stapfia 65 Biologiezentrum, Oberösterreichisches Landesmuseum, Linz.

Vöth W (2000) *Gymnadenia, Nigritella* und ihre Bestäuber. Jour Eur Orch 32: 547-573.

Wang XJ (2009) Pollination biology of *Dendrobium fimbriatum* and *Coelogyne viscosa* (Orchidaceae). Master’s Thesis. Beijing: Graduate University of Chinese Academy of Sciences. [in Chinese]

Wilckman H (2011) The split-apply-combine strategy for data analysis. J Stat Softw 40:1-29. <https://www.jstatsoft.org/article/view/v040i01>

Wickham H (2016) ggplot2: elegant graphics for data analysis. Springer-Verlag, New York. ISBN 978-3-319-24277-4.

Wilcove DS, Rothstein D, Dubow J, Phillips A, Losos E (1998) Quantifying threats to imperiled species in the United States. BioScience 48:607-615. <https://doi.org/10.2307/1313420>

Wroblewska A, Szczepaniak L, Bajguz A, Jedrzejczyk I, Talalaj I, Ostrowiecka B, Brzosko E, Jermakowicz E, Mirski P (2019) Deceptive strategy in *Dactylorhiza* orchids: multidirectional evolution of floral chemistry. Ann Bot 123: 1005-1016. <https://doi.org/10.1093/aob/mcz003>

Yang JQ (2017) Pollination observations on wild *Cymbidium faberi*. South China Agriculture 18: 38-39. [In Chinese]

Yu XH, Luo YB, Dong M (2008) Pollination biology of *Cymbidium goeringii* (Orchidaceae) in China. J Syst Evol 46: 163-174. <https://doi.org/10.3724/SP.J.1002.2008.06203>

Zha ZB, Tang J, Liang YL, Ding H, Luo HL, Yang BY (2016) Breeding system and pollination biology of *Goodyera foliosa* (Orchidaceae). Trop Subtrop Bot 24:333-341.

Zhou X, Lin H, Fan XL, Gao JY (2012) Autonomous self-pollination and insect visitation in a saprophytic orchid, *Epipogium roseum* (D. Don) Lindl. Aust J Bot 60:154-159. <https://doi.org/10.1071/BT11265>

Zhou X, Liu Q, Han JY, Gao JY (2016) Different pollinator assemblages ensure reproductive success of *Cleisostoma linearilobatum* (Orchidaceae) in fragmented holy hill forest and traditional tea garden. Sci Rep 6: 21435. <https://doi.org/10.1038/srep21435>
